# Glycocalyx-targeted therapy prevents age-related muscle loss and declines in maximal exercise capacity

**DOI:** 10.18632/aging.206313

**Published:** 2025-08-30

**Authors:** Daniel R. Machin, Md. Torikul Islam, Alec Malouf, Daniel Nguyen, Mostafa Sabouri, Maryana Boulos, Andrew G. Horn, Kiana M. Schulze, Gwenael Layec, Lisa A. Lesniewski, Anthony J. Donato

**Affiliations:** 1 Department of Cell Biology and Physiology, Vascular Physiology Group, University of New Mexico School of Medicine, Albuquerque, NM 87131, USA; 2 Department of Internal Medicine, University of Utah, Salt Lake City, UT 84112, USA; 3 Department of Nutrition and Integrative Physiology, University of Utah, Salt Lake City, UT 84112, USA; 4 Department of Health and Kinesiology, University of Nebraska Omaha, Omaha, NE 68182, USA; 5 Department of Biochemistry, University of Utah, Salt Lake City, UT 84112, USA; 6 GRECC, VA Salt Lake City, Salt Lake City, UT 84112, USA; 7 Nora Eccles Harrison Cardiovascular Research and Training Institute, University of Utah, Salt Lake City, UT 84112, USA

**Keywords:** glycocalyx, aging, hyaluronan

## Abstract

Age-related declines in cardiovascular function contribute to reduced physical capacity, both of which are independent predictors of mortality. We have previously demonstrated that glycocalyx-targeted therapy with Endocalyx™ that contains high-molecular-weight hyaluronan (HMW-HA) improves cardiovascular health in old age, raising the possibility that HMW-HA also plays a role in age-related physical dysfunction. Here, we first demonstrate that tamoxifen-inducible deletion of *Has2*, which produces HMW-HA, leads to glycocalyx depletion, decreases exercise capacity, and impairs skeletal muscle respiratory capacity. We then sought to determine the effects of Endocalyx™ on physical function in old mice. Young (7 months) and old (29 months) mice underwent standard diet or Endocalyx^™^-supplemented diet for 10 weeks. Glycocalyx thickness was higher in young and Endocalyx^™^-treated old mice compared to standard diet-fed old mice. While standard diet-fed old mice demonstrated a reduction in running exercise capacity over the intervention, Endocalyx^™^-supplemented diet prevented this age-related decline. Gastrocnemius citrate synthase activity, a marker of mitochondrial content in skeletal muscle, was lower in standard diet-fed old mice compared to young and Endocalyx^™^-treated old mice. Collectively, these findings suggest that glycocalyx integrity is a critical determinant of physical function and that glycocalyx-targeted interventions may be a viable therapeutic strategy to treat age-related physical dysfunction.

## INTRODUCTION

Advancing age is accompanied by functional declines within many physiological systems, notably the vasculature and skeletal musculature. Maximal exercise capacity is a powerful predictor of mortality [1–3], and its gradual age-related decline is largely attributed to loss of cardiovascular function [4–7] and skeletal muscle function [8]. The endothelial glycocalyx is a gel-like structure bound to the vascular endothelium that is critical for a healthy vasculature [9]. The glycocalyx directly participates in endothelium-dependent dilation by mechanotransducing shear stress to the endothelium, stimulating nitric oxide-mediated vasodilation [10, 11]. The glycocalyx also passively modulates flow resistance to regulate blood flow homogeneity throughout the microvasculature [12, 13]. Hyaluronan is one of the primary glycosaminoglycans that comprise the endothelial glycocalyx. Although there is no consensus on whether hyaluronan quantity decreases across the lifespan [14, 15], it has been demonstrated that the hyaluronan molecular weight profile shifts from high molecular weight- hyaluronan (≥500 kDa (HMW-HA)) in youth to low molecular weight-hyaluronan in advanced age [16, 17]. The importance of maintaining a youthful hyaluronan molecular weight profile is demonstrated by observations that HMW-HA has anti-aging, vasoprotective, and cancer-resistant properties [18, 19]. Indeed, it is understood that the HMW-HA produced by naked mole rats, which is five times larger than HMW-HA produced by mice or humans, is partially responsible for their extraordinary longevity [18].

We have shown that the glycocalyx is degraded in advanced age [20], as well as in several disease states [9, 21–23]. Moreover, in advanced age there is an age-related reduction in expression of hyaluronan synthase 2 (*Has2*) [20], which is the hyaluronan synthase isoform that produces the majority of HMW-HA in mammals [24]. Given that skeletal muscle is a highly metabolic tissue that requires an intact microvasculature for appropriate nutrient delivery [6, 25, 26], it is possible that age-related glycocalyx degradation may be an underlying contributor to microvascular dysfunction, loss of skeletal muscle mass, and, ultimately, loss of exercise capacity. We have previously shown that Endocalyx^™^, a glycocalyx-targeted therapy that contains HMW-HA, restores the endothelial glycocalyx and ameliorates age-related microvascular and arterial dysfunction [27]. This raises the possibility that Endocalyx^™^ can be an effective treatment to prevent age-related declines in maximal exercise capacity. Therefore, we sought to examine several markers of physiological functional capacity mice following the induction of *Has2* deletion, as well as in old mice that receive Endocalyx^™^ treatment. We hypothesized that, compared to wild-type mice, *Has2* deletion would have lower glycocalyx thickness that was accompanied by a decrease in maximal exercise capacity. Moreover, we further hypothesized that compared to age-matched control mice, old mice that receive Endocalyx^™^ treatment will have restored glycocalyx properties that are accompanied by higher maximal exercise capacity and skeletal muscle mass.

## RESULTS

### Has2 deletion impairs maximal exercise capacity and mitochondrial respiration

**Figure 1:**
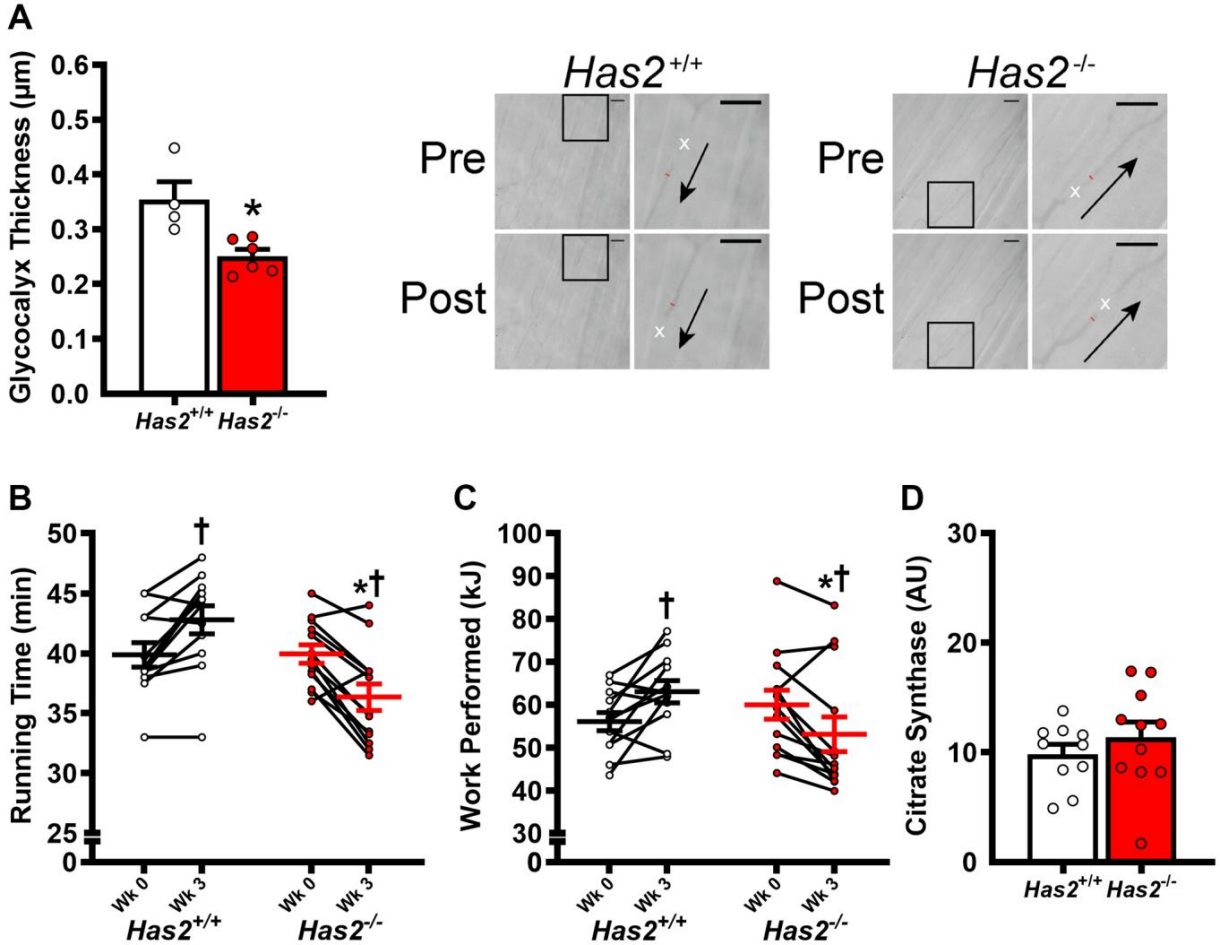
**Impact of *Has2* deletion on glycocalyx integrity and exercise capacity.** (**A**) Glycocalyx thickness in gastrocnemius muscle microcirculation prior to tamoxifen injections (Wk 0) and 3 weeks post-tamoxifen administration (Wk 3) with representative images demonstrating changes in perfused diameter (red line segment) pre- and post-leukocyte (white x’s) passage with arrows representing direction of flow; scale bar = 50 μm. (**B**) Running time to exhaustion during maximal treadmill test pre- and post-tamoxifen administration. (**C**) Work performed during maximal treadmill test pre- and post-tamoxifen administration. (**D**) Gastrocnemius muscle citrate synthase activity. Data are presented as mean ± SEM. ^*^P < 0.05 vs. *Has2*^+/+^; ^†^*P* < 0.05 vs. Pre. *N* = 4–13/group.

**Figure 2:**
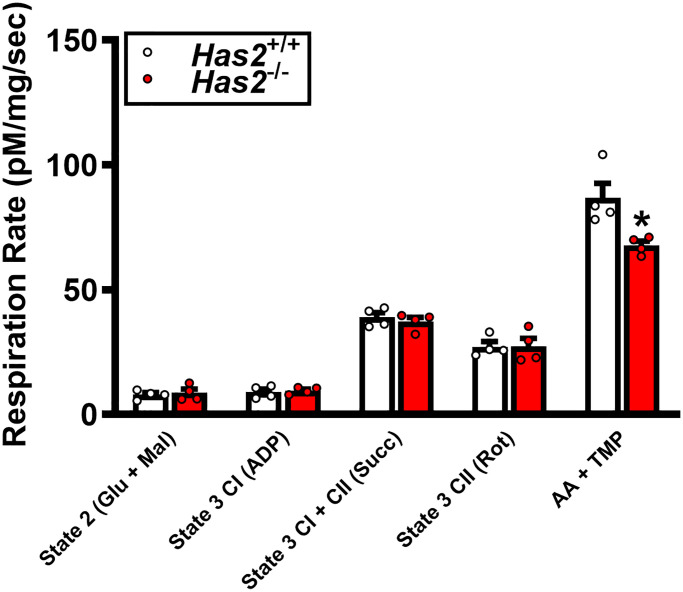
**Mitochondrial respiration in gastrocnemius muscle of *Has2*^+/+^ and *Has2*^−/−^ mice post-tamoxifen administration.** Respiration rates were measured under various substrate conditions: State 2 (Glu + Mal), State 3 CI (ADP), State 3 CI + CII (Succ), State 3 CII (Rot), and AA + TMPD. Data are presented as mean ± SEM. ^*^P < 0.05 vs. *Has2*^+/+^. *N* = 4–5/group.

We have previously shown that tamoxifen-induced *Has2* deletion results in a 75% knockdown of arterial *Has2* mRNA expression in *Has2*^−/−^ mice compared with *Has2*^+/+^ mice [27]. Here, we sought to examine the effects of *Has2* deletion on skeletal muscle microcirculatory glycocalyx thickness and exercise capacity. Glycocalyx thickness measured within the gastrocnemius microcirculation was lower in *Has2*^−/−^ compared to *Has2*^+/+^ mice (Figure 1A; *P* < 0.05), indicating that *Has2* deletion led to a reduction in glycocalyx properties of skeletal muscle. We observed no change in body mass of *Has2*^+/+^ (Pre: 24.7 ± 1.0 vs. Post: 24.9 ± 0.8 g; *P* > 0.05) or *Has2*^−/−^ (Pre: 26.3 ± 1.3 vs. Post: 26.6 ± 1.0 g; *P* > 0.05) mice prior to and following tamoxifen administration. Tissue masses of internal organs and skeletal muscles were also similar between *Has2*^−/−^ or *Has2*^+/+^ mice (Table 1; *P* > 0.05). Compared to *Has2*^−/−^, *Has2*^+/+^ mice had a greater maximal exercise capacity, as determined by running time to exhaustion and work performed during the maximal treadmill running test following tamoxifen administration (Figure 1B, 1C, respectively; *P* < 0.05 for both). Running time was similar between groups prior to tamoxifen administration (*P* > 0.05). Both *Has2*^−/−^ and *Has2*^+/+^ mice demonstrated a change in running time and work performed prior to and following tamoxifen administration (*P* < 0.05). Indeed, running time and work performed increased in *Has2*^+/+^ mice during that time period (*P* < 0.05), whereas *Has2*^−/−^ mice demonstrated a decrease in running time and work performed (*P* < 0.05). Citrate synthase activity, measured in the gastrocnemius muscle, was similar between *Has2*^−/−^ and *Has2*^+/+^ mice (Figure 1D; *P* > 0.05). Mitochondrial respiration rates in state 2 and state 3 (ADP-stimulated respiration) in the gastrocnemius muscle were similar between *Has2*^−/−^ and *Has2*^+/+^ mice (Figure 2; *P* > 0.05), while complex IV-specific maximal oxygen respiration was blunted in *Has2*^−/−^ compared to *Has2*^+/+^ mice (*P* < 0.05). Taken together, these results suggest that the glycocalyx is an upstream regulator of skeletal muscle function and can be targeted therapeutically to improve physical function in old age.

**Table 1:** Characteristics of *Has2*^+/+^ and *Has2*^−/−^ mice.

	***Has2* ^+/+^**	***Has2* ^−/−^**
Age, mo	5.1 ± 0.2	5.2 ± 0.2
Heart, mg	123 ± 3	126 ± 5
Heart/body mass, mg/g	4.92 ± 0.11	5.10 ± 0.15
Liver, mg	1,261 ± 103	1,154 ± 98
Liver/body mass, mg/g	49.87 ± 3.36	46.70 ± 3.81
Quadriceps, mg	190 ± 11	191 ± 10
Quadriceps/body mass, mg/g	7.55 ± 0.36	7.68 ± 0.24
Gastrocnemius, mg	133 ± 13	133 ± 6
Gastrocnemius/body mass, mg/g	5.31 ± 0.50	5.37 ± 0.19
Soleus, mg	10 ± 0	10 ± 0
Soleus/body mass, mg/g	0.40 ± 0.01	0.39 ± 0.02

Data are mean ± SEM. *N* = 12–14/group.

### Age-related declines in body mass and skeletal muscle mass are prevented by glycocalyx-targeted therapy

**Figure 3:**
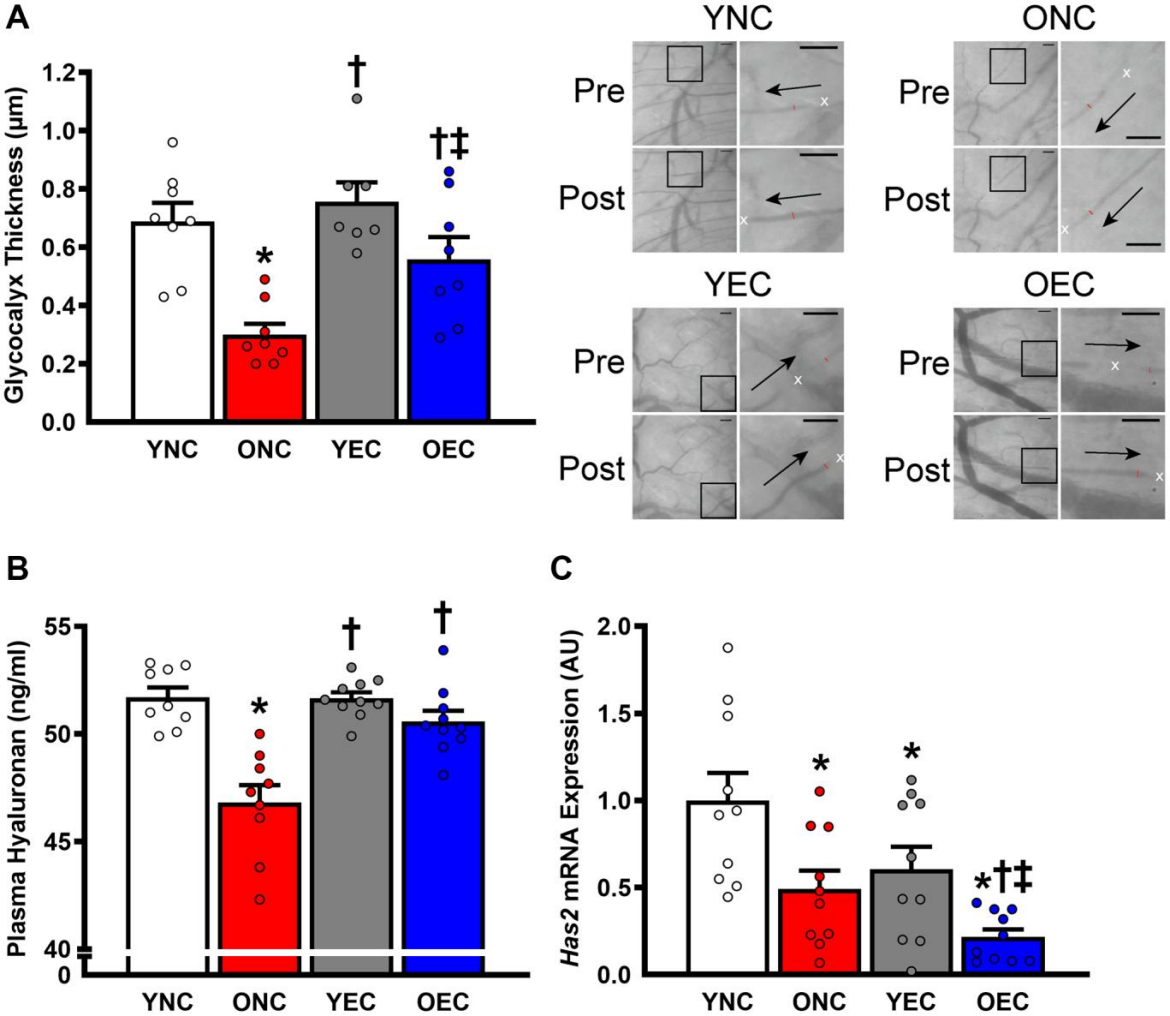
**Glycocalyx integrity in young normal chow (YNC), old normal chow (ONC), young Endocalyx^™^ treatment (YEC), and old Endocalyx^™^ treatment (OEC) mice.** (**A**) Glycocalyx thickness with representative images demonstrating changes in perfused diameter (red line segment) pre- and post-leukocyte (white x’s) passage with arrows representing direction of flow; scale bar = 50 μm. (**B**) Plasma hyaluronan concentrations. (**C**) Aortic *Has2* mRNA expression. Data are presented as mean ± SEM. ^*^P < 0.05 vs. YNC; ^†^*P* < 0.05 vs. ONC; ^‡^*P* < 0.05 vs. YEC; *N* = 7–10/group.

**Figure 4:**
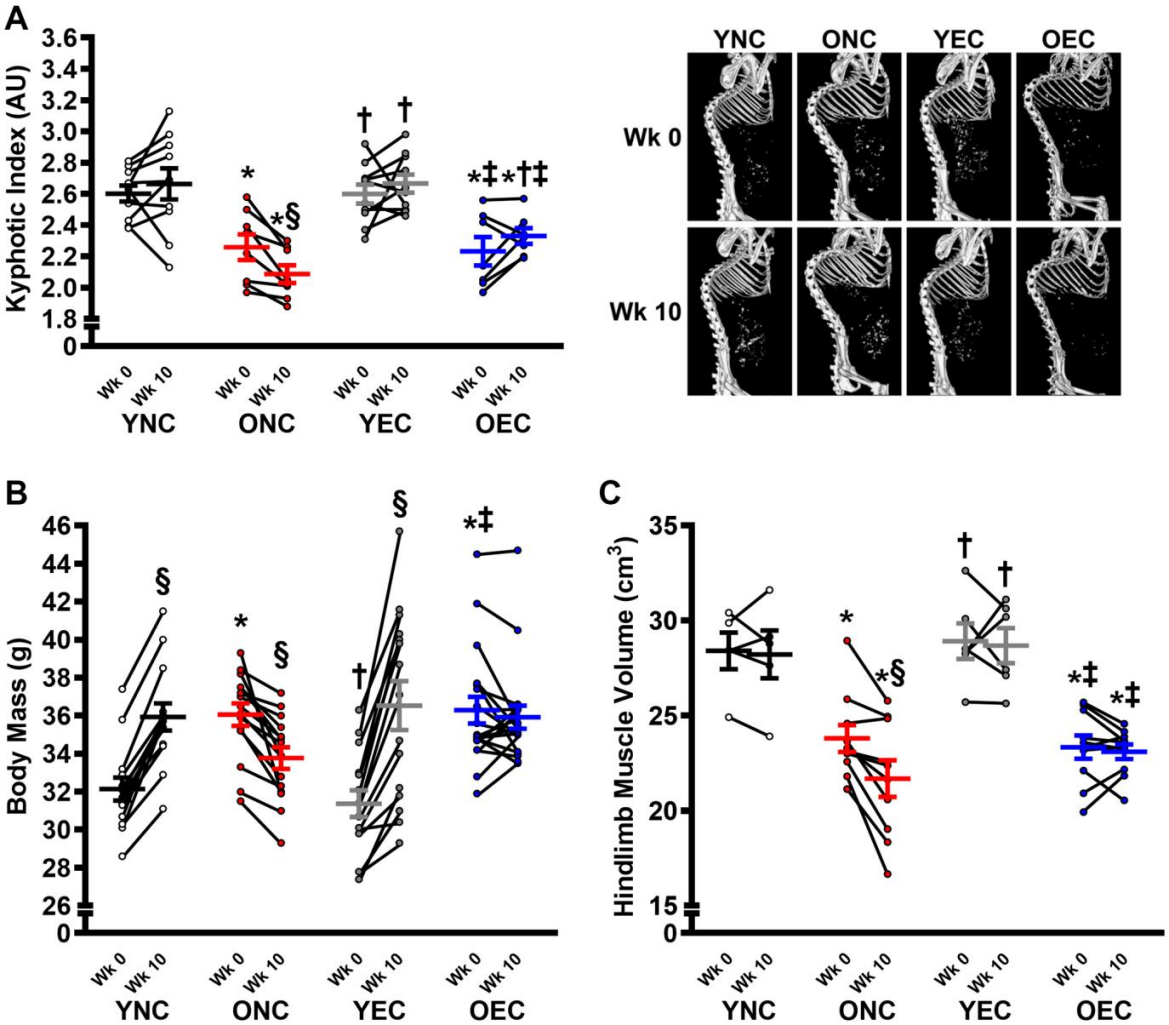
**Frailty, body mass, and muscle characteristics in young normal chow (YNC), old normal chow (ONC), young Endocalyx^™^ treatment (YEC), and old Endocalyx^™^ treatment (OEC) mice.** (**A**) Kyphosis index at Week 0 and Week 10 with representative images. (**B**) Body mass at Week 0 and Week 10. (**C**) Hindlimb muscle volume at Week 0 and Week 10. Data are presented as mean ± SEM. ^*^P < 0.05 vs. YNC; ^†^*P* < 0.05 vs. ONC; ^‡^*P* < 0.05 vs. YEC; ^§^*P* < 0.05 vs. Week 0. *N* = 5–15/group.

Dietary intervention length and caloric intake were similar between young normal chow (YNC), old normal chow (ONC), young Endocalyx^™^ treatment (YEC), and old Endocalyx^™^ treatment (OEC) mice (Table 2; *P* > 0.05). We observed 4 unexpected mortalities in ONC mice and 2 unexpected mortalities in OEC mice during the intervention. Young and old mice that received glycocalyx-targeted restorative therapy with Endocalyx^™^ treatment had a greater consumption of HMW-HA than age-matched controls (Table 2; *P* < 0.05). Glycocalyx thickness and plasma hyaluronan were higher in YNC, YEC, and OEC compared to ONC mice (Figure 3A, 3B, respectively; *P* < 0.05). Aortic *Has2* mRNA expression was higher in YNC mice compared to all other groups (Figure 3C; *P* < 0.05). ONC and YEC mice had similar *Has2* expression (*P* > 0.05), which was higher than OEC mice (*P* < 0.05). Compared to ONC and OEC mice, YNC and YEC mice had a higher kyphosis index, indicating lower frailty at Week 0 and 10 (Figure 4A; *P* < 0.05). We observed a decrease in kyphosis index from Week 0 to 10 in ONC mice (*P* < 0.05). There was no change in kyphosis index in YNC, YEC, and OEC mice between Week 0 and 10 (*P* > 0.05). Compared to ONC and OEC mice, YNC and YEC mice had a lower body mass at Week 0 (Figure 4B; *P* < 0.05). After the 10-week dietary intervention YNC and YEC mice increased body mass (*P* < 0.05), while ONC mice decreased body mass (*P* < 0.05 Table 2/Figure 4B). OEC mice maintained their body mass demonstrating no change (*P* > 0.05). At week 10, body mass was similar between YNC, YEC, and OEC mice (*P* > 0.05), while there was a tendency for body mass to be lower in ONC compared to YNC and OEC (*P* = 0.07–0.11). Tissue masses also differed between groups. Heart and liver masses were higher in ONC and OEC compared to YNC and YEC mice, visceral adipose tissue and hindlimb skeletal muscle (excluding soleus) masses were lower in ONC and OEC compared to YNC and YEC mice (Table 2; *P* < 0.05 for all). While tissue masses were similar within age groups (*P* > 0.05), OEC mice had greater visceral adipose and hindlimb skeletal muscle masses compared to ONC mice (*P* < 0.05). Compared to ONC and OEC mice, YNC and YEC mice had a higher hindlimb skeletal muscle volume at Week 0 and 10 (Figure 4C; *P* < 0.05). Hindlimb muscle volume did not differ within age groups at Week 0 or 10 (*P* > 0.05), although there was reduction in muscle volume in ONC mice from Week 0 to 10 (*P* < 0.05). YNC, YEC, and OEC mice demonstrated no change in muscle volume from Week 0 to 10 (*P* > 0.05).

**Table 2:** Characteristics of young and old B6D2F1/J mice.

	**YNC**	**ONC**	**YEC**	**OEC**
Age, mo	7.4 ± 0.1	29.9 ± 0.1^*^	7.3 ± 0.1^†^	29.7 ± 0.1^*‡^
Caloric intake, kcal/day	12.7 ± 0.0	12.9 ± 0.4	12.9 ± 0.2	12.9 ± 0.1
HMW-HA intake, μg/day	0.0 ± 0.0	0.0 ± 0.0	36.9 ± 0.5^*†^	36.9 ± 0.3^*†^
Total Cholesterol, mg/dL	75.8 ± 4.1	71.3 ± 4.4	65.2 ± 3.1	69.4 ± 5.1
HDL Cholesterol, mg/dL	50.4 ± 2.5	44.5 ± 2.7	46.3 ± 2.7	41.8 ± 2.9^*^
LDL Cholesterol, mg/dL	2.1 ± 0.5	4.5 ± 0.4^*^	1.9 ± 0.3^†^	4.2 ± 0.4^*‡^
Triglycerides, mg/dL	116.4 ± 13.0	111.5 ± 13.9	85.4 ± 6.4	117.0 ± 19.7
Glucose, mg/dL	148.6 ± 5.7	136.6 ± 7.2	138.5 ± 4.2	134.6 ± 5.1
Heart, mg	186 ± 4	234 ± 8^*^	177 ± 4^†^	228 ± 7^*‡^
Heart/body mass, mg/g	5.11 ± 0.12	6.69 ± 0.22^*^	4.89 ± 0.11^†^	6.26 ± 0.21^*‡^
Liver, mg	1,681 ± 34	1,871 ± 103	1,609 ± 60^†^	1,958 ± 98^*‡^
Liver/body mass, mg/g	46.23 ± 1.18	53.35 ± 2.78^*^	44.11 ± 0.93^†^	53.48 ± 2.66^*‡^
Visceral adipose tissue, mg	1,021 ± 95	231 ± 28^*^	989 ± 108^†^	555 ± 138^*‡^
Visceral adipose tissue/body mass, mg/g	27.82 ± 2.29	6.43 ± 0.71^*^	26.54 ± 2.34^†^	14.38 ± 3.12^*‡^
Quadriceps, mg	261 ± 5	157 ± 6^*^	261 ± 7^†^	187 ± 7^*†‡^
Quadriceps/body mass, mg/g	7.18 ± 0.20	4.51 ± 0.18^*^	7.21 ± 0.24^†^	5.12 ± 0.19^*†‡^
Gastrocnemius, mg	192 ± 4	114 ± 9^*^	183 ± 6^†^	146 ± 6^*†‡^
Gastrocnemius/body mass, mg/g	5.29 ± 0.18	3.28 ± 0.26^*^	5.07 ± 0.19^†^	3.98 ± 0.15^*†‡^
Soleus, mg	12 ± 1	11 ± 1	11 ± 1	12 ± 1
Soleus/body mass, mg/g	0.33 ± 0.02	0.30 ± 0.02	0.30 ± 0.02	0.34 ± 0.02
Plantaris, mg	26 ± 2	14 ± 1^*^	26 ± 1^†^	19 ± 1^*†‡^
Plantaris/body mass, mg/g	0.71 ± 0.04	0.41 ± 0.03^*^	0.66 ± 0.06^†^	0.52 ± 0.03^*†‡^

^*^P < 0.05 vs. YNC. ^†^*P* < 0.05 vs. ONC. ^‡^*P* < 0.05 vs. YEC. Data are mean ± SEM. *N* = 14–19/group. Abbreviations: YNC: Young normal chow; ONC: Old normal chow; YEC: Young Endocalyx^™^; OEC: Old Endocalyx^™^.

### The age-related decline in maximal exercise capacity is prevented by glycocalyx-targeted therapy

**Figure 5:**
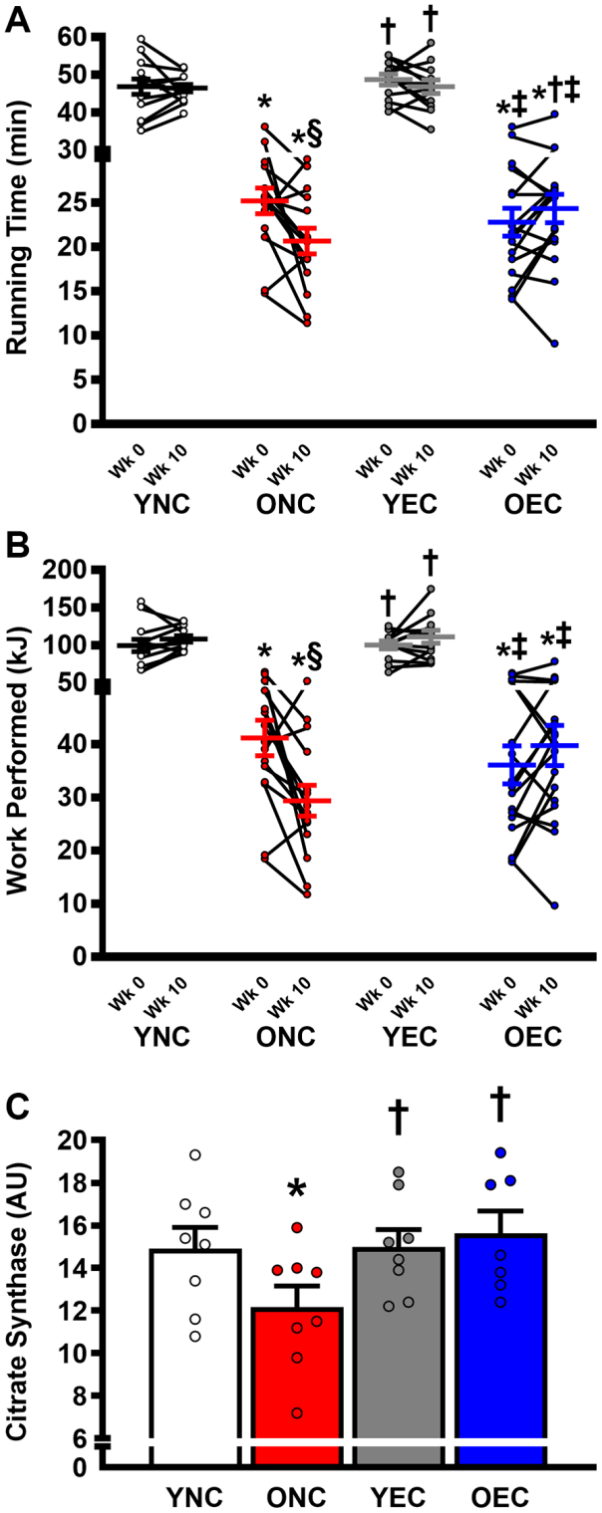
**Exercise capacity and muscle oxidative function young normal chow (YNC), old normal chow (ONC), young Endocalyx^™^ treatment (YEC), and old Endocalyx^™^ treatment (OEC) mice.** (**A**) Running time to exhaustion during maximal treadmill test at Week 0 and Week 10. (**B**) Work performed during maximal treadmill test at Week 0 and Week 10. (**C**) Gastrocnemius muscle citrate synthase activity. Data are presented as mean ± SEM. ^*^P < 0.05 vs. YNC; ^†^*P* < 0.05 vs. ONC; ^‡^*P* < 0.05 vs. YEC; ^§^*P* < 0.05 vs. Week 0. *N* = 7–13/group.

Compared to ONC and OEC, YNC and YEC mice had a greater maximal exercise capacity, as determined by running time to exhaustion and work performed during the maximal treadmill running test at Week 0 and 10 (Figure 5A, 5B, respectively; *P* < 0.05 for both). Running time to exhaustion was similar between ONC and OEC at Week 0 (*P* > 0.05). In contrast, at Week 10, running time was higher in OEC compared to ONC mice (*P* < 0.05). Indeed, ONC mice demonstrated a reduction in running time to exhaustion and work performed over the 10-week dietary intervention period (*P* < 0.05), while no change was detected in YNC, YEC, and OEC mice over that time-period (*P* > 0.05). Citrate synthase activity, measured in the gastrocnemius muscle, was higher in YNC, YEC, and OEC compared to ONC mice (Figure 5C; *P* < 0.05). Citrate synthase activity was similar between YNC, YEC, and OEC mice (*P* > 0.05).

## DISCUSSION

The present study sought to determine the impact of HMW-HA on maximal exercise capacity and skeletal muscle mass and function. To do so, we investigated the effects of induced, whole body *Has2* deletion in young mice. Inducible *Has2* deletion resulted in lower gastrocnemius glycocalyx thickness that was accompanied by a decrease in maximal exercise capacity. Although skeletal muscle mass was not different between *Has2*^−/−^ and *Has2*^+/+^ mice, complex IV-specific mitochondrial respiratory capacity was impaired following induction of *Has2* deletion. To study the effects of glycocalyx restoration on age-related declines in maximal exercise capacity and skeletal muscle function, we investigated effects of Endocalyx^™^, a glycocalyx-targeted therapy that contains HMW-HA, on markers of physiological functional capacity in old mice. We observed that Endocalyx^™^ prevented the continued progression of frailty and loss of body mass in old mice. Importantly, further declines in maximal exercise capacity were prevented in old mice that received Endocalyx^™^. The benefits of Endocalyx^™^ were accompanied by a restored endothelial glycocalyx, maintenance of skeletal muscle mass, and greater skeletal muscle mitochondrial content assessed by citrate synthase activity. Taken together, these findings suggest manipulations to the endothelial glycocalyx may impact maximal exercise capacity by altering skeletal muscle properties.

### Whole body Has2 deletion impairs maximal exercise capacity and mitochondrial function

To determine the role of HMW-HA on skeletal muscle mass and maximal exercise capacity, we generated inducible, whole body *Has2* knockout mice. We and other have shown that *Has2* deletion can reduce glycocalyx properties and impair markers of arterial health throughout the circulatory system [27, 28]. We have also shown that induced *Has2* deletion phenocopies aging, indicated by impaired larger artery function. Given the role of *Has2* on both the endothelial glycocalyx and larger artery function, we would expect that the dysfunction would occur in other systems as a result of induced *Has2* deletion. Indeed, in the present study, we observed a reduction in maximal exercise capacity in *Has2*^−/−^ mice 3-weeks post-tamoxifen treatment. This reduction in exercise capacity appeared to be independent of changes in body mass or skeletal muscle mass. Although there were no differences in skeletal muscle mass between *Has2*^−/−^ and *Has2*^+/+^ mice, we did observe slightly impaired mitochondrial function in skeletal muscle. Interestingly, we also observed greater citrate synthase activity in old mice that received Endocalyx^™^ treatment. While it is possible that augmented glycocalyx properties could improve nutrient delivery to skeletal muscle at rest and during exercise, it is also possible that HMW-HA plays other roles that are integral to skeletal muscle health and function [29]. Nevertheless, future study is warranted to determine the direct role that the glycocalyx plays in skeletal muscle health.

### Endocalyx^™^ prevents further frailty and declines in skeletal muscle mass and maximal exercise capacity

Maximal exercise capacity is a powerful predictor of mortality [1–3], and its gradual age-related decline is largely attributed to declines in cardiovascular [4–7] and skeletal muscle function [8]. As expected, at the start of the intervention, old mice were in substantially worse physical condition than young mice. Although body mass was higher in old mice, they had lower hindlimb muscle volume and maximal exercise capacity. Although Endocalyx^™^ did not restore markers of frailty, skeletal muscle mass, or maximal exercise capacity to values similar to young mice, it did appear to stop further progression of frailty and loss of skeletal muscle volume and maximal exercise capacity in old mice. Indeed, at the end of the dietary intervention, old mice that received Endocalyx^™^ were less frail and had greater muscle mass and maximal exercise capacity than age-matched control mice.

The mechanism by which Endocalyx^™^ conferred these benefits to old mice requires further investigation. We have previously shown that this Endocalyx^™^ restores the endothelial glycocalyx and ameliorates age-related microvascular and arterial dysfunction in old mice [27]. In the microvasculature, the glycocalyx modulates flow resistance to regulate blood flow homogeneity through microvessels, such as arterioles [12, 13]. Given that skeletal muscle is a highly metabolic tissue that requires an intact microvasculature for appropriate nutrient delivery [6, 25, 26], it is likely that age-related microvascular dysfunction and loss of skeletal muscle mass are interrelated and that glycocalyx deterioration may be an underlying contributor by impairing nutrient delivery to skeletal muscles. Thus, it is possible that the benefits observed in the present study are related to a restored glycocalyx and improved microvascular function.

We also treated young mice with Endocalyx^™^. Although they seemingly had no benefit of this treatment on frailty, skeletal muscle properties, or maximal exercise capacity, there was also no benefit of glycocalyx-targeted therapies on the glycocalyx, as glycocalyx thickness was similar between YNC, YEC, and OEC mice in this study. It’s unclear why glycocalyx thickness did not increase in young mice, as we and others have previously shown that a Western diet is capable of improving glycocalyx properties in young mice [30–32]. It should be noted that our group has also shown that despite a thicker glycocalyx, a Western diet still induces arterial dysfunction [33]. The lack of observed increase in glycocalyx thickness in YEC may be attributed to unchanged plasma hyaluronan concentrations. HMW-HA is a primary component of both the glycocalyx and is an active ingredient in Endocalyx^™^. Interestingly, in both young and old mice that received Endocalyx^™^ treatment, we saw lower arterial *Has2* gene expression. *Has2* is responsible for producing the vast majority of HMW-HA in mammals, thus, it is possible that hyaluronan levels were maintained in YEC mice via negative feedback, which led to *Has2* downregulation.

### Perspectives

It is well known that maximal exercise capacity decreases with advancing age [34–39], but can be increased by endurance exercise training at nearly any age [40, 41] and in many disease conditions [42]. In many cases, increases in maximal exercise capacity are accompanied by improvements in cardiovascular function [41, 43–46]. Moreover, individuals that maintain endurance training with advancing age have greater maximal exercise capacity and cardiovascular function than age-matched sedentary controls [47, 48]. Still, even in highly trained individuals that maintain training volume/intensity, maximal exercise capacity still declines with advancing age, but does remain higher than age-matched sedentary individuals [36, 49–51]. It is possible that preserved maximal exercise capacity and skeletal muscle mass and function in old mice that received Endocalyx^™^ treatment was achieved through increases in physical activity. However, it should be noted that dietary intake was similar between control and Endocalyx^™^-treated mice. Regardless, if the benefits of Endocalyx^™^ are achieved by increased physical activity, improved peripheral hemodynamics as a result of glycocalyx restoration is the likely underlying mechanism. Future studies are warranted to determine the precise mechanisms by which these improvements occurred.

### Limitations and future directions

As with any study there are limitations. For the experiments involving Endocalyx^™^ treatment, only male mice were studied. Although female mice were not included, it is likely that we would have observed a similar benefit of Endocalyx^™^ treatment. Indeed, a recent study that only used female mice observed improvements in glycocalyx properties following 10 weeks of Endocalyx^™^ treatment in a diabetic mouse model [52]. Moreover, our experiments in male and female *Has2*^−/−^ mice demonstrate proof-of-concept that lower glycocalyx thickness as a result of induced *Has2* deletion are accompanied by reductions in maximal exercise capacity in both sexes. We observed a reduction in maximal exercise capacity in *Has2*^−/−^ mice following tamoxifen injections, while maximal exercise capacity in *Has2*^+/+^ mice was improved over the same timeframe. Baseline treadmill test was performed at 3.6 ± 0.4 mo with the post-test occurring ~3 weeks after tamoxifen treatment at 4.3 ± 0.5 mo. Although these mice did not gain body mass during this time, they likely continued to mature physically, which may explain why *Has2*^+/+^ mice had improved maximal exercise capacity. Future studies examine the effects of *Has2* deletion should be performed in mice that have physically matured to a level where exercise capacity is stable over the period before and after tamoxifen treatment. Moreover, future studies are warranted to determine if Endocalyx^™^ treatment is capable of restoring maximal exercise capacity in *Has2*^−/−^ mice. The results of the present study are largely focused on the endothelial glycocalyx. However, nearly every cell-type grows a glycocalyx. Although the glycocalyx of skeletal muscle fiber and smooth muscle cells are less understood, future studies are warranted to determine if they play a role in age-related dysfunction observed in the present study.

## CONCLUSIONS

We have previously shown that age-related microvascular and arterial dysfunction can be ameliorated by Endocalyx^™^, a glycocalyx-targeted therapy that contains HMW-HA. In the present study we sought to extend those findings by showing that induced, whole body *Has2* deletion was accompanied by a decrease in maximal exercise capacity and mitochondrial function. Furthermore, we identified that Endocalyx^™^ prevented the continued progression of frailty and loss of body mass in old mice. Importantly, further declines in maximal exercise capacity were prevented in old mice that Endocalyx^™^ treatment and were accompanied by a maintenance of skeletal muscle mass and increased skeletal muscle mitochondrial content. Taken together, these findings provide direct evidence of a role for HMW-HA in the modulation of exercise capacity.

## METHODS

### Ethical approval

All animal procedures conformed to the Guide to the Care and Use of Laboratory Animals: Eighth Edition [53] and were approved by the University of Utah (protocol #: 16-02002) and Veteran’s Affairs Medical Center-Salt Lake City (protocol #: A16/16) Animal Care and Use Committees.

### Animals

All mice were group-housed in standard mouse cages in an animal care facility at the VAMC-SLC on a 12:12 light:dark cycle. Food and water were supplied *ad libitum* in group housed cages.

Conditional whole-body deletion of *Has2* (exon 2 deletion) was used to manipulate glycocalyx properties in young male and female mice (4–5 mo) [27, 54]. Mice that expressed Cre-recombinase in all tissues under control of the tamoxifen-inducible estrogen receptor T2 moiety were crossed with mice homozygous for floxed *Has2* (*Has2*^F/F^) and wild type *Has2* (*Has2*^+/+^) to create compound *Has2*^F/F^ + CreER and *Has2*^+/+^ + CreER mice for use as *Has2* deficient experimental animals and wild-type controls, respectively. *Has2*^−/−^ mice were generated by inducing Cre-recombinase-mediated deletion of *Has2*^F/F^ in all tissues of *Has2*^F/F^ + CreER mice with daily intraperitoneal injections of tamoxifen (2 mg/day) for a total of 5 days. Importantly, both *Has2*^F/F^ + CreER mice and *Has2*^+/+^ + CreER wild-type control mice were injected with identical tamoxifen treatment regimens to control for the effect of tamoxifen in all subsequent analyses (here-after tamoxifen treated animals are denoted as *Has2*^−/−^ and *Has2*^+/+^ mice). *Has2*^−/−^ and *Has2*^+/+^ mice were derived from the same litters to ensure that the final experimental and wild-type control mice had identical genetic backgrounds. Mice were fed a normal chow control diet (Envigo, Teklad Diet #8604; Protein: 24.3%, Carbohydrate 40.9%, Fat 4.7% by kcal).

Young male B6D2F1/J mice were purchased from Jackson Laboratories and old male B6D2F1/J mice were obtained from the aging colonies maintained at Charles River Inc. for the National Institute on Aging. Mice were provided 4 weeks to acclimate to the animal care facility at the VAMC-SLC before beginning the study. Mice were fed either a normal chow control diet (Envigo, Teklad Diet #8604; Protein: 24.3%, Carbohydrate 40.9%, Fat 4.7% by kcal) or glycocalyx precursor supplemented diet (37 mg/kg of glucosamine sulfate, fucoidan, superoxide dismutase, and HMW-HA (Endocalyx^™^, Microvascular Health Solutions LLC, Alpine, UT, USA) in Envigo, Teklad Diet #8604; provided courtesy of MicroVascular Health Solutions, LLC (U.S. Patent Serial No. 9,943,572)) for 10 weeks [27].

### Intravital microscopy

The mesenteric (B6D2F1 mice) or gastrocnemius (*Has2* mice) microcirculations were observed using intravital microscopy, as described previously [20, 30]. Glycocalyx thickness was derived by measuring the change in perfused diameter immediately before and after the passage of a spontaneous leukocyte in individual microvessels, as described previously [30, 55, 56].

### Quantitative PCR

mRNA expression was measured in the aorta of mice by quantitative PCR. Briefly, RNA isolated from aortic tissue was used to synthesize cDNA via QuantiTect Reverse Transcription kit (Qiagen, Inc., Valencia, CA, USA). Quantitative PCR was performed using RT^2^ SYBR^®^ Green quantitative PCR Mastermix (Qiagen, Inc.). Fold change in mRNA expression was calculated as the fold difference in expression of target mRNA to 18s rRNA 2^−(target C_T_ − 18s C_T_)^ and normalized to young values. *18s* primer sequences: forward: TAGAGGGACAAGTGGCGTTC; reverse: CGCTGAGCCAGTCAGTGT. *Has2* primer sequences: forward: TCAGCGAAGTTATGGGCAGG; reverse: TCTGTCTCACCAGGTCCCTT.

### Maximal exercise capacity

A graded exercise test was performed, as described previously [57], on a rodent treadmill (Columbus Instruments, Columbus, OH, USA) to assess maximal exercise capacity prior to and after the 10-week dietary intervention. The exercise test was performed at a 25° incline and consisted of a 5 min warm up at 5 m/min followed by increases in treadmill speed of 1 m/min every 3 min thereafter until exhaustion. Throughout the entire test, the tails of the mice were agitated with a brush when they ran toward or fell off the rear of the treadmill. Exhaustion was determined as a mouse that could not climb back on the treadmill to continue running, despite tail agitation. A familiarization session to treadmill running was performed on the day prior to the graded exercise test with mice running at 25° incline for 10 min at 5 m/min, 5 min at 7 m/min, and 1 min at 10 m/min.

### Mitochondrial respiration

The tissue preparation and respiration measurement techniques were adapted from established methods [58, 59] and have been previously described [60]. Briefly, BIOPS-immersed fibers were carefully separated with fine-tip forceps and subsequently bathed in a BIOPS-based saponin solution (50 μg/ml) for 30 minutes. Following saponin treatment, muscle fibers were rinsed twice in ice-cold mitochondrial respiration fluid (MiR05, in mM: 110 Sucrose, 0.5 EGTA, 3 MgCl_2_, 60 K-lactobionate, 20 taurine, 10 KH_2_PO_4_, 20 HEPES, BSA 1 g.L^−1^, pH 7.1) for 10 minutes each. After the muscle sample was gently dabbed with a paper towel to remove excess fluid, the wet weight of the sample was measured using a standard, calibrated scale (2–4 mg). The muscle fibers were then placed in the respiration chamber (Oxygraph O2K, Oroboros Instruments, Innsbruck, Austria) with 2 ml of MiR05 solution and warmed to 37°C. After allowing the permeabilized muscle sample to equilibrate for 5 minutes, mitochondrial respiratory function was assessed in duplicate. To assess the function of each mitochondrial complex, O_2_ consumption was assessed with the addition of a series of respiratory substrates and inhibitors in the following order and final concentrations in the chamber: glutamate-malate (10 and 2 mM), ADP (5 mM), succinate (10 mM), cytochrome c (10 μM), rotenone (0.5 μM), antimycin-A (2.5 μM), oligomycin (2 μM), and N,N,N,N-tetramethyl-p-phenylenediamine (TMPD)-ascorbate (2 and 0.5 mM). Pilot studies indicated that the concentration of the substrates and inhibitors used were at saturating levels [60]. This allowed the determination of (1) state 2 respiration, the non-phosphorylating resting state, which provides an index of proton leak, assessed in the presence of malate + glutamate; (2) complex-I driven state 3 respiration (State 3_CI_), the ADP-activated state of oxidative phosphorylation, assessed in the presence of glutamate + malate + ADP; (3) complex I + II driven state 3 respiration (state 3_CI+CII_), assessed in the presence of glutamate + malate + ADP + succinate; (4) complex II driven state 3 respiration (state 3_CII_), assessed in the presence of glutamate + malate + ADP + succinate + rotenone, and (5) uncoupled_CIV_ respiration, where the link between the electron transport chain and ATP synthesis has been abolished, assessed by inhibiting complex III (antimycin A) and complex V (oligomycin) followed by the addition of TMPD + ascorbate.

### Lipid profile and glucose

Total cholesterol, total triglycerides, and LDL and high-density lipoprotein (HDL) concentrations were measured using dedicated on-board reagents from Abbott Laboratories (Cat#7D62-21, 7D74-21, 1E31-20, and 3k33-22) [61]. Blood glucose was measured prior to sacrifice using a Precision Xceed Pro Glucometer in blood collected via a tail nick.

### Plasma hyaluronan

Plasma concentrations of HA were measured by HA Quantikine ELISA kit (R&D Systems, Minneapolis, MN, USA) according to the manufacturer’s protocol.

### Computerized tomography

Mice were anesthetized with isoflurane (2–3%) in 100% oxygen at 2 L/min flow rate and scanned using a Quantum FX Micro CT Scanner (Perkin-Elmer, Waltham, MA, USA). Voltage and current were set at 50 kV and 200 μA, respectively, and the images were captured over a 4.5 min interval. Analysis was conducted with Caliper Analyze 11.0 (Analyze Direct, Overland Park, KS, USA). Kyphotic index (KI) was used as a determinant of frailty in anesthetized mice using Micro-CT. Briefly, KI in mice was calculated from Micro-CT images in the sagittal plane using the equation: KI = AB/CD, as described previously [62]. Line AB was drawn from the posterior edge of C7 to the posterior edge of L6. Line CD is the perpendicular distance from line AB to the dorsal border of the vertebral body farthest from line AB. KI was calculated from left and right sagittal views and averaged. Hindlimb muscle volumes were assessed using a small animal computerized tomography (Micro-CT), as described previously [63].

### Citrate synthase activity

Snap-frozen gastrocnemius samples were homogenized with homogenization buffer (250 mM sucrose, 40 mM KCl, 2 mM EGTA, 20 mM Tris-HCl; Sigma Aldrich, St. Louis, MO, USA). Citrate synthase activity was assessed via spectrophotometry (Synergy 4; Biotek Instruments, Winooski, VT, USA) [64].

### Statistical analysis

Statistics were performed using GraphPad Prism (IBM, Chicago, IL, USA). Student’s *t*-tests, one-way ANOVA, or two-way repeated measures ANOVA were used to evaluate differences between groups. When a significant ANOVA was present, the two-stage step-up method of Benjamini, Krieger, and Yekutieli was used to identify values that were significantly different. Statistical significance was set at *P* < 0.05 for all analyses. Data are presented as mean ± SEM.

### Data availability

The data that support the findings of this study are available on request from the corresponding author.

## References

[1] Myers J, Prakash M, Froelicher V, Do D, Partington S, Atwood JE. Exercise capacity and mortality among men referred for exercise testing. N Engl J Med. 2002; 346:793–801. 10.1056/NEJMoa011858 11893790

[2] Mora S, Redberg RF, Cui Y, Whiteman MK, Flaws JA, Sharrett AR, Blumenthal RS. Ability of exercise testing to predict cardiovascular and all-cause death in asymptomatic women: a 20-year follow-up of the lipid research clinics prevalence study. JAMA. 2003; 290:1600–7. 10.1001/jama.290.12.1600 14506119

[3] Blair SN, Kohl HW 3rd, Paffenbarger RS Jr, Clark DG, Cooper KH, Gibbons LW. Physical fitness and all-cause mortality. A prospective study of healthy men and women. JAMA. 1989; 262:2395–401. 10.1001/jama.262.17.2395 2795824

[4] Fleg JL, O'Connor F, Gerstenblith G, Becker LC, Clulow J, Schulman SP, Lakatta EG. Impact of age on the cardiovascular response to dynamic upright exercise in healthy men and women. J Appl Physiol (1985). 1995; 78:890–900. 10.1152/jappl.1995.78.3.890 7775334

[5] Behnke BJ, Barstow TJ, Kindig CA, McDonough P, Musch TI, Poole DC. Dynamics of oxygen uptake following exercise onset in rat skeletal muscle. Respir Physiol Neurobiol. 2002; 133:229–39. 10.1016/s1569-9048(02)00183-0 12425970

[6] Behnke BJ, Delp MD, Dougherty PJ, Musch TI, Poole DC. Effects of aging on microvascular oxygen pressures in rat skeletal muscle. Respir Physiol Neurobiol. 2005; 146:259–68. 10.1016/j.resp.2004.12.009 15766914

[7] Behnke BJ, Kindig CA, Musch TI, Koga S, Poole DC. Dynamics of microvascular oxygen pressure across the rest-exercise transition in rat skeletal muscle. Respir Physiol. 2001; 126:53–63. 10.1016/s0034-5687(01)00195-5 11311310

[8] Fleg JL, Lakatta EG. Role of muscle loss in the age-associated reduction in VO2 max. J Appl Physiol (1985). 1988; 65:1147–51. 10.1152/jappl.1988.65.3.1147 3182484

[9] Machin DR, Phuong TT, Donato AJ. The role of the endothelial glycocalyx in advanced age and cardiovascular disease. Curr Opin Pharmacol. 2019; 45:66–71. 10.1016/j.coph.2019.04.011 31112922 PMC7055464

[10] Yao Y, Rabodzey A, Dewey CF Jr. Glycocalyx modulates the motility and proliferative response of vascular endothelium to fluid shear stress. Am J Physiol Heart Circ Physiol. 2007; 293:H1023–30. 10.1152/ajpheart.00162.2007 17468337

[11] Pries AR, Secomb TW, Gaehtgens P. The endothelial surface layer. Pflugers Arch. 2000; 440:653–66. 10.1007/s004240000307 11007304

[12] McClatchey PM, Schafer M, Hunter KS, Reusch JE. The endothelial glycocalyx promotes homogenous blood flow distribution within the microvasculature. Am J Physiol Heart Circ Physiol. 2016; 311:H168–76. 10.1152/ajpheart.00132.2016 27199117 PMC6189750

[13] Pries AR, Secomb TW. Microvascular blood viscosity in vivo and the endothelial surface layer. Am J Physiol Heart Circ Physiol. 2005; 289:H2657–64. 10.1152/ajpheart.00297.2005 16040719

[14] Reed MJ, Vernon RB, Damodarasamy M, Chan CK, Wight TN, Bentov I, Banks WA. Microvasculature of the Mouse Cerebral Cortex Exhibits Increased Accumulation and Synthesis of Hyaluronan With Aging. J Gerontol A Biol Sci Med Sci. 2017; 72:740–6. 10.1093/gerona/glw213 28482035 PMC6075594

[15] Yannariello-Brown J, Chapman SH, Ward WF, Pappas TC, Weigel PH. Circulating hyaluronan levels in the rodent: effects of age and diet. Am J Physiol. 1995; 268:C952–7. 10.1152/ajpcell.1995.268.4.C952 7733243

[16] Kamada H, Masuda K, D'Souza AL, Lenz ME, Pietryla D, Otten L, Thonar EJ. Age-related differences in the accumulation and size of hyaluronan in alginate culture. Arch Biochem Biophys. 2002; 408:192–9. 10.1016/s0003-9861(02)00543-x 12464271

[17] Holmes MW, Bayliss MT, Muir H. Hyaluronic acid in human articular cartilage. Age-related changes in content and size. Biochem J. 1988; 250:435–41. 10.1042/bj2500435 3355532 PMC1148875

[18] Tian X, Azpurua J, Hine C, Vaidya A, Myakishev-Rempel M, Ablaeva J, Mao Z, Nevo E, Gorbunova V, Seluanov A. High-molecular-mass hyaluronan mediates the cancer resistance of the naked mole rat. Nature. 2013; 499:346–9. 10.1038/nature12234 23783513 PMC3720720

[19] Neumann A, Schinzel R, Palm D, Riederer P, Münch G. High molecular weight hyaluronic acid inhibits advanced glycation endproduct-induced NF-kappaB activation and cytokine expression. FEBS Lett. 1999; 453:283–7. 10.1016/s0014-5793(99)00731-0 10405161

[20] Machin DR, Bloom SI, Campbell RA, Phuong TTT, Gates PE, Lesniewski LA, Rondina MT, Donato AJ. Advanced age results in a diminished endothelial glycocalyx. Am J Physiol Heart Circ Physiol. 2018; 315:H531–9. 10.1152/ajpheart.00104.2018 29750566 PMC6172638

[21] Machin DR, Gates PE, Vink H, Frech TM, Donato AJ. Automated Measurement of Microvascular Function Reveals Dysfunction in Systemic Sclerosis: A Cross-sectional Study. J Rheumatol. 2017; 44:1603–11. 10.3899/jrheum.170120 28916547 PMC5668162

[22] Bloom SI, Tucker JR, Machin DR, Abdeahad H, Adeyemo AO, Thomas TG, Bramwell RC, Lesniewski LA, Donato AJ. Reduction of double-strand DNA break repair exacerbates vascular aging. Aging (Albany NY). 2023; 15:9913–47. 10.18632/aging.205066 37787989 PMC10599741

[23] Morgan RG, Walker AE, Trott DW, Machin DR, Henson GD, Reihl KD, Cawthon RM, Denchi EL, Liu Y, Bloom SI, Phuong TT, Richardson RS, Lesniewski LA, Donato AJ. Induced Trf2 deletion leads to aging vascular phenotype in mice associated with arterial telomere uncapping, senescence signaling, and oxidative stress. J Mol Cell Cardiol. 2019; 127:74–82. 10.1016/j.yjmcc.2018.11.014 30502348 PMC7216296

[24] Itano N, Sawai T, Yoshida M, Lenas P, Yamada Y, Imagawa M, Shinomura T, Hamaguchi M, Yoshida Y, Ohnuki Y, Miyauchi S, Spicer AP, McDonald JA, Kimata K. Three isoforms of mammalian hyaluronan synthases have distinct enzymatic properties. J Biol Chem. 1999; 274:25085–92. 10.1074/jbc.274.35.25085 10455188

[25] Muller-Delp JM, Spier SA, Ramsey MW, Delp MD. Aging impairs endothelium-dependent vasodilation in rat skeletal muscle arterioles. Am J Physiol Heart Circ Physiol. 2002; 283:H1662–72. 10.1152/ajpheart.00004.2002 12234821

[26] Behnke BJ, Ramsey MW, Stabley JN, Dominguez JM 2nd, Davis RT 3rd, McCullough DJ, Muller-Delp JM, Delp MD. Effects of aging and exercise training on skeletal muscle blood flow and resistance artery morphology. J Appl Physiol (1985). 2012; 113:1699–708. 10.1152/japplphysiol.01025.2012 23042906 PMC3544508

[27] Machin DR, Trott DW, Gogulamudi VR, Islam MT, Bloom SI, Vink H, Lesniewski LA, Donato AJ. Glycocalyx-targeted therapy ameliorates age-related arterial dysfunction. Geroscience. 2023; 45:2351–65. 10.1007/s11357-023-00745-1 36787090 PMC10651573

[28] van den Berg BM, Wang G, Boels MGS, Avramut MC, Jansen E, Sol WMP, Lebrin F, van Zonneveld AJ, de Koning EJP, Vink H, Gröne HJ, Carmeliet P, van der Vlag J, Rabelink TJ. Glomerular Function and Structural Integrity Depend on Hyaluronan Synthesis by Glomerular Endothelium. J Am Soc Nephrol. 2019; 30:1886–97. 10.1681/ASN.2019020192 31308073 PMC6779367

[29] Calve S, Isaac J, Gumucio JP, Mendias CL. Hyaluronic acid, HAS1, and HAS2 are significantly upregulated during muscle hypertrophy. Am J Physiol Cell Physiol. 2012; 303:C577–88. 10.1152/ajpcell.00057.2012 22785117 PMC3468343

[30] Zheng X, Deacon C, King AJ, Machin DR. Microcirculatory and glycocalyx properties are lowered by high-salt diet but augmented by Western diet in genetically heterogeneous mice. Am J Physiol Heart Circ Physiol. 2022; 322:H328–35. 10.1152/ajpheart.00656.2021 34995168 PMC8799391

[31] Mitsuda S, Uzawa K, Sawa M, Ando T, Yoshikawa T, Miyao H, Yorozu T, Ushiyama A. Vascular Endothelial Glycocalyx Plays a Role in the Obesity Paradox According to Intravital Observation. Front Cardiovasc Med. 2021; 8:727888. 10.3389/fcvm.2021.727888 34796208 PMC8593246

[32] Sabouri M, Li Z, Zheng X, Berg Sen J, Bernardo J, Machin D. Identifying the time course and contributors for Western diet-induced increase in glycocalyx barrier function. Physiol. 2024; 39:595. 10.1152/physiol.2024.39.S1.595

[33] Zheng X, Li Z, Berg Sen J, Samarah L, Deacon CS, Bernardo J, Machin DR. Western diet augments metabolic and arterial dysfunction in a sex-specific manner in outbred, genetically diverse mice. Front Nutr. 2023; 9:1090023. 10.3389/fnut.2022.1090023 36687716 PMC9853899

[34] Pimentel AE, Gentile CL, Tanaka H, Seals DR, Gates PE. Greater rate of decline in maximal aerobic capacity with age in endurance-trained than in sedentary men. J Appl Physiol (1985). 2003; 94:2406–13. 10.1152/japplphysiol.00774.2002 12533496

[35] Tanaka H, Desouza CA, Jones PP, Stevenson ET, Davy KP, Seals DR. Greater rate of decline in maximal aerobic capacity with age in physically active vs. sedentary healthy women. J Appl Physiol (1985). 1997; 83:1947–53. 10.1152/jappl.1997.83.6.1947 9390967

[36] Fitzgerald MD, Tanaka H, Tran ZV, Seals DR. Age-related declines in maximal aerobic capacity in regularly exercising vs. sedentary women: a meta-analysis. J Appl Physiol (1985). 1997; 83:160–5. 10.1152/jappl.1997.83.1.160 9216959

[37] Wilson TM, Tanaka H. Meta-analysis of the age-associated decline in maximal aerobic capacity in men: relation to training status. Am J Physiol Heart Circ Physiol. 2000; 278:H829–34. 10.1152/ajpheart.2000.278.3.H829 10710351

[38] Robinson S. Experimental studies of physical fitness in relation to age. Arbeitsphysiologie. 1938; 10:251–323. 10.1007/BF02011412

[39] Loe H, Rognmo Ø, Saltin B, Wisløff U. Aerobic capacity reference data in 3816 healthy men and women 20-90 years. PLoS One. 2013; 8:e64319. 10.1371/journal.pone.0064319 23691196 PMC3654926

[40] Badenhop DT, Cleary PA, Schaal SF, Fox EL, Bartels RL. Physiological adjustments to higher-or lower-intensity exercise in elders. Med Sci Sports Exerc. 1983; 15:496–502. 6656559

[41] Tanaka H, Dinenno FA, Monahan KD, Clevenger CM, DeSouza CA, Seals DR. Aging, habitual exercise, and dynamic arterial compliance. Circulation. 2000; 102:1270–5. 10.1161/01.cir.102.11.1270 10982542

[42] Alkatan M, Baker JR, Machin DR, Park W, Akkari AS, Pasha EP, Tanaka H. Improved Function and Reduced Pain after Swimming and Cycling Training in Patients with Osteoarthritis. J Rheumatol. 2016; 43:666–72. 10.3899/jrheum.151110 26773104

[43] Alkatan M, Machin DR, Baker JR, Akkari AS, Park W, Tanaka H. Effects of Swimming and Cycling Exercise Intervention on Vascular Function in Patients With Osteoarthritis. Am J Cardiol. 2016; 117:141–5. 10.1016/j.amjcard.2015.10.017 26541906

[44] Whelton SP, Chin A, Xin X, He J. Effect of aerobic exercise on blood pressure: a meta-analysis of randomized, controlled trials. Ann Intern Med. 2002; 136:493–503. 10.7326/0003-4819-136-7-200204020-00006 11926784

[45] Cornelissen VA, Smart NA. Exercise training for blood pressure: a systematic review and meta-analysis. J Am Heart Assoc. 2013; 2:e004473. 10.1161/JAHA.112.004473 23525435 PMC3603230

[46] Seals DR, Tanaka H, Clevenger CM, Monahan KD, Reiling MJ, Hiatt WR, Davy KP, DeSouza CA. Blood pressure reductions with exercise and sodium restriction in postmenopausal women with elevated systolic pressure: role of arterial stiffness. J Am Coll Cardiol. 2001; 38:506–13. 10.1016/s0735-1097(01)01348-1 11499745

[47] Vaitkevicius PV, Fleg JL, Engel JH, O'Connor FC, Wright JG, Lakatta LE, Yin FC, Lakatta EG. Effects of age and aerobic capacity on arterial stiffness in healthy adults. Circulation. 1993; 88:1456–62. 10.1161/01.cir.88.4.1456 8403292

[48] Tanaka H, DeSouza CA, Seals DR. Absence of age-related increase in central arterial stiffness in physically active women. Arterioscler Thromb Vasc Biol. 1998; 18:127–32. 10.1161/01.atv.18.1.127 9445266

[49] Eskurza I, Donato AJ, Moreau KL, Seals DR, Tanaka H. Changes in maximal aerobic capacity with age in endurance-trained women: 7-yr follow-up. J Appl Physiol (1985). 2002; 92:2303–8. 10.1152/japplphysiol.01124.2001 12015340

[50] Pollock ML, Mengelkoch LJ, Graves JE, Lowenthal DT, Limacher MC, Foster C, Wilmore JH. Twenty-year follow-up of aerobic power and body composition of older track athletes. J Appl Physiol (1985). 1997; 82:1508–16. 10.1152/jappl.1997.82.5.1508 9134900

[51] Heath GW, Hagberg JM, Ehsani AA, Holloszy JO. A physiological comparison of young and older endurance athletes. J Appl Physiol Respir Environ Exerc Physiol. 1981; 51:634–40. 10.1152/jappl.1981.51.3.634 7327965

[52] Smith JA, Ramirez-Perez FI, Burr K, Gonzalez-Vallejo JD, Morales-Quinones M, McMillan NJ, Ferreira-Santos L, Sharma N, Foote CA, Martinez-Lemus LA, Padilla J, Manrique-Acevedo C. Impact of dietary supplementation of glycocalyx precursors on vascular function in type 2 diabetes. J Appl Physiol (1985). 2024; 137:1592–603. 10.1152/japplphysiol.00651.2024 39480270 PMC11687847

[53] Guide for the Care and Use of Laboratory Animals. National Research Council (US) Committee for the Update of the Guide for the Care and Use of Laboratory Animals, 8th edition. Washington (DC): National Academies Press; 2011. ISBN-13: 978-0-309-15400-0, ISBN-10: 0-309-15400-6. 21595115

[54] Matsumoto K, Li Y, Jakuba C, Sugiyama Y, Sayo T, Okuno M, Dealy CN, Toole BP, Takeda J, Yamaguchi Y, Kosher RA. Conditional inactivation of Has2 reveals a crucial role for hyaluronan in skeletal growth, patterning, chondrocyte maturation and joint formation in the developing limb. Development. 2009; 136:2825–35. 10.1242/dev.038505 19633173 PMC2730409

[55] Nieuwdorp M, Meuwese MC, Mooij HL, Ince C, Broekhuizen LN, Kastelein JJ, Stroes ES, Vink H. Measuring endothelial glycocalyx dimensions in humans: a potential novel tool to monitor vascular vulnerability. J Appl Physiol (1985). 2008; 104:845–52. 10.1152/japplphysiol.00440.2007 18162484

[56] Liuhanen S, Sallisalmi M, Pettilä V, Oksala N, Tenhunen J. Indirect measurement of the vascular endothelial glycocalyx layer thickness in human submucosal capillaries with a plug-in for ImageJ. Comput Methods Programs Biomed. 2013; 110:38–47. 10.1016/j.cmpb.2012.10.019 23195494

[57] Phuong TTT, Walker AE, Henson GD, Machin DR, Li DY, Donato AJ, Lesniewski LA. Deletion of Robo4 prevents high-fat diet-induced adipose artery and systemic metabolic dysfunction. Microcirculation. 2019; 26:e12540. 10.1111/micc.12540 30825241 PMC7217325

[58] Pesta D, Gnaiger E. High-resolution respirometry: OXPHOS protocols for human cells and permeabilized fibers from small biopsies of human muscle. Methods Mol Biol. 2012; 810:25–58. 10.1007/978-1-61779-382-0_3 22057559

[59] Kuznetsov AV, Veksler V, Gellerich FN, Saks V, Margreiter R, Kunz WS. Analysis of mitochondrial function in situ in permeabilized muscle fibers, tissues and cells. Nat Protoc. 2008; 3:965–76. 10.1038/nprot.2008.61 18536644

[60] Gifford JR, Trinity JD, Layec G, Garten RS, Park SY, Rossman MJ, Larsen S, Dela F, Richardson RS. Quadriceps exercise intolerance in patients with chronic obstructive pulmonary disease: the potential role of altered skeletal muscle mitochondrial respiration. J Appl Physiol (1985). 2015; 119:882–8. 10.1152/japplphysiol.00460.2015 26272320 PMC4610006

[61] Islam MT, Hall SA, Dutson T, Bloom SI, Bramwell RC, Kim J, Tucker JR, Machin DR, Donato AJ, Lesniewski LA. Endothelial cell-specific reduction in mTOR ameliorates age-related arterial and metabolic dysfunction. Aging Cell. 2024; 23:e14040. 10.1111/acel.14040 38017701 PMC10861194

[62] Laws N, Hoey A. Progression of kyphosis in mdx mice. J Appl Physiol (1985). 2004; 97:1970–7. 10.1152/japplphysiol.01357.2003 15234960

[63] Islam MT, Henson GD, Machin DR, Bramwell RC, Donato AJ, Lesniewski LA. Aging differentially impacts vasodilation and angiogenesis in arteries from the white and brown adipose tissues. Exp Gerontol. 2020; 142:111126. 10.1016/j.exger.2020.111126 33203620 PMC8407014

[64] Gifford JR, Trinity JD, Kwon OS, Layec G, Garten RS, Park SY, Nelson AD, Richardson RS. Altered skeletal muscle mitochondrial phenotype in COPD: disease vs. disuse. J Appl Physiol (1985). 2018; 124:1045–53. 10.1152/japplphysiol.00788.2017 29357496 PMC5972462

